# Evaluation of the gamified application KIJANI to promote physical activity in children and adolescents: A multimethod study

**DOI:** 10.1177/20552076241271861

**Published:** 2024-08-18

**Authors:** Laura Willinger, Florian Schweizer, Birgit Böhm, Daniel A Scheller, Stephan Jonas, Renate Oberhoffer-Fritz, Jan Müller, Lara Marie Reimer

**Affiliations:** 1 Preventive Pediatrics, 9184 Technical University of Munich, Munich, Germany; 2 School of Computation, 663294 Information and Technology, 9184 Technical University of Munich, Munich, Germany; 3 Didactics in Sport and Health, 9184 Technical University of Munich, Munich, Germany; 4 Institute for Digital Medicine, 39062 University Hospital Bonn, Bonn, Germany

**Keywords:** Physical activity, health promotion, digital health, gamification

## Abstract

**Objective:**

Digital approaches have the potential to make activity promotion attractive and age-appropriate for children and adolescents. KIJANI is a mobile application aiming to increase physical activity (PA) in youth via gamification and augmented reality. This study investigates the user experience with KIJANI through a multimethod approach.

**Approaches:**

KIJANI is based on the concept that virtual coins can be earned through PA, for example, in the form of collected step counts. With these coins, blocks can be bought, which can be used to create virtual buildings and landscapes and integrate these into the player's real-world environment via augmented reality. To evaluate the user experience, participants played KIJANI in groups of three for 25 min. Afterwards KIJANI was evaluated qualitatively with one-on-one semi-structured interviews as well as quantitatively with standardized questionnaires.

**Results:**

Overall, 22 participants (12.6 ± 1.7 years, 6 girls) were included in the study. The overall game concept and realization were well received by the target group. Study participants did have various and creative ideas for the further development of KIJANI. The majority (*n *= 16) thought that using KIJANI would increase their PA level. User experience based on the UEQ scale was (mean ± SD): attractiveness (1.78 ± 1.82), perspicuity (2.15 ± 0.680), efficiency (0.67 ± 1.25), dependability, (1.21 ± 0.93), stimulation (1.24 ± 1.78), and novelty (1.27 ± 1.34).

**Conclusion:**

With these insights, a further step has been taken in the participatory development of KIJANI. Apps like KIJANI appear to be suitable for PA promotion in children and adolescents.

## Introduction

Physical inactivity has emerged as a widespread global health concern, with its roots often taking hold during childhood and adolescence.^1,2^ In Germany, only 26% of children and adolescents meet the recommendations of the World Health Organization (WHO) of engaging in a minimum of 60 min of moderate-to-vigorous physical activity (PA) on average per day.^3,4^ Despite the well-established multifaceted advantages associated with regular PA, encompassing enhanced cardiometabolic health, bone health, cognitive performance, and mental well-being^5,6^ the prevalence of physical inactivity in the younger population remains a remarkable challenge.

Recognizing the critical role of adolescence as a pivotal transitional phase, during which foundational health habits and future health behaviors are established and endured into adulthood,^7–9^ it is compelling to promote PA during this developmental stage. Adolescents, being avid users of digital devices, present a unique opportunity to leverage digital technology for age-appropriate and appealing prevention and health promotion strategies.^4^ In response to this, we have developed a digital intervention approach named KIJANI, specifically designed to promote PA in this age group. KIJANI is a smartphone application that aims to motivate users through a game-based concept to engage in physical movement particularly in outdoor settings. The potential of mobile health interventions to elevate PA levels among children and adolescents has been shown by prior research.^10^ Various studies have demonstrated that especially gamification in health applications positively influences the behavior of the target population.^11–13^ Through a playful context, gamification has been shown to empower children and adolescents to attain recommended activity goals.^14^ Gamified interventions benefit from various psychological mechanisms. Gamification can transparently illustrate goals and their relevance, nudge users toward long-term engagement, provide direct feedback, offer positive reinforcement, and simplify content. Additionally, gamification facilitates social comparison and connection among users, enabling them to support each other and work toward a common goal.^15^ These underlying psychological factors align with behavior change theories, which form the foundation of our work aimed at encouraging children and adolescents to adopt a physically active lifestyle. Especially the fusion of virtual and real-world elements in the form of augmented reality has huge potential to enhance PA and social interaction.^16^ A previous study showed that the integration of augmented reality into exergames is especially attractive for children.^17^ In light of these insights, this study aims to investigate the user experience of children and adolescents using the KIJANI app through a multimethod approach.

## Methods

### Study design and participants

This study used a multimethod design in which the user experience of the KIJANI app was evaluated with the target group. The multimethod approach leverages the strengths of both qualitative and quantitative research, offering a more comprehensive and reliable understanding of the suitability of user-centered interventions like KIJANI.^18^

Study participants used the KIJANI app for 25 min in a group of three participants at a KIJANI play location under the supervision of the researcher. Smartphones with the pre-installed KIJANI app were provided by the researcher. The researcher ensured that all participants of a group have sufficient time and experience with the app allowing them to give informed feedback at a later stage. Afterwards, the app was evaluated qualitatively with each participant in one-on-one semi-structured interviews as well as quantitatively with standardized questionnaires on user experience, PA enjoyment as well as activity-related self-efficacy, all paper-pencil. Permissions to use the questionnaires were obtained from the copyright holders.

With this methodology, a participatory design approach is implemented, as continuous user involvement in the development of mHealth applications is strongly recommended by previous research. Interviews have been demonstrated to be a prevalent evaluation method within user-centered design processes.^19^

Children and adolescents at the age of 10–16 years were included in this study between August and December 2023 in Germany, in order to evaluate the experience with KIJANI during different seasons. Children with walking impairments and cognitive impairments that limit their ability to understand the task were excluded from the study. The KIJANI app is designed to be accessible to children and young people from different backgrounds. To ensure inclusivity, the study incorporated participants both with and without chronic illnesses, as well as from various socioeconomic backgrounds.

Twelve participants were recruited via a summer sports camp for children and adolescents with and without chronic conditions. Five of these participants did have congenital heart disease, two were in surveillance after pediatric oncological disease, one participant had a chronic respiratory disease, and four participants were free of any diagnosed chronic condition. Ten study participants were included via a social support institution for children and adolescents. All eligible participants that were recruited agreed to be part of the study. During the intervention and interviews, the responsible persons of the respective institution were present, but was not involved in the data collection.

This study was conducted in accordance with the Declaration of Helsinki and Good Clinical Practice. The study protocol was approved by the ethical board of the Technical University of Munich (project number: 2023-185-S-NP). All children and their guardians provided written informed consent.

### KIJANI intervention

KIJANI stands as a German abbreviation for “Children & Youth: active, nature-conscious, innovative!.” KIJANI is a smartphone application explicitly developed for children and adolescents with the aim to increase PA through a gamified approach. A detailed description of the KIJANI app can be found in the study protocol (DOI: 10.2196/55156), in the following only a short description is displayed.

**Figure 1.**
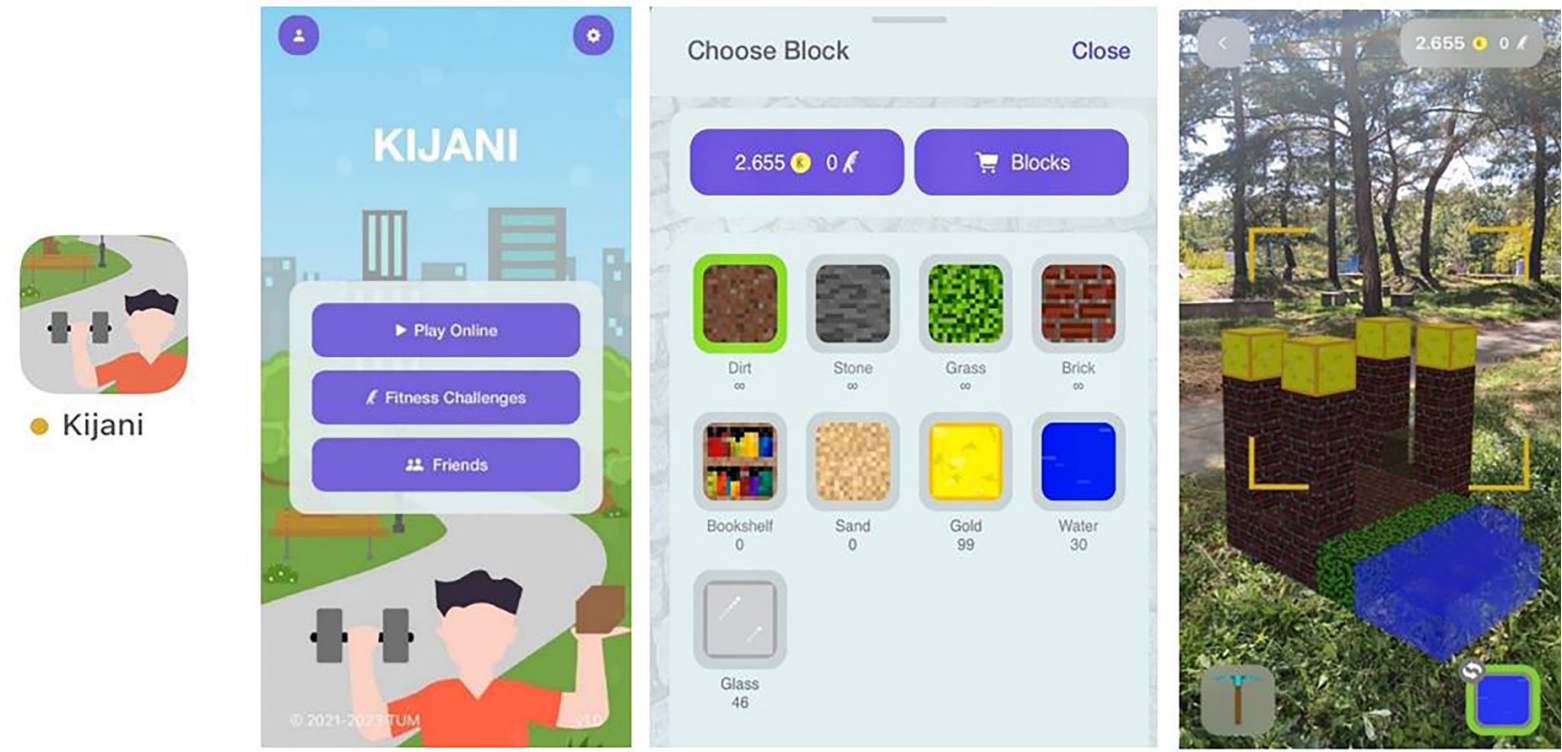
KIJANI app (left), KIJANI screen (left middle), KIJANI blocks (right middle), KIJANI game (right).

The basic game concept is that virtual coins can be “earned” through PA, for example, in the form of collected step count. These coins can in turn be used to “buy” blocks in the KIJANI app, which can be used to create virtual buildings and integrate them into the player's real-world environment via augmented reality, see Figure 1. PA of users is detected via accelerometers integrated into the smartphones using Apple's HealthKit as the data source. KIJANI accesses the PA data represented by steps via the Apple HealthKit interface.

KIJANI is designed to be played both alone and with friends in a group. In addition, the individual activity behavior can be compared with friends in a ranking list, which is another incentive for PA.^20^ KIJANI can only be played in comprehensively defined, child-friendly locations that were identified in the partner project “WALKI-MUC” through participatory methods. When selecting the play locations through participatory methods, special attention was paid to safety and attractiveness of the locations to ensure an optimal gaming experience. This ensures that KIJANI can only be used in a safe and appealing outdoor environment for the target group. There is a location search feature implemented in the KIJANI app to find the play locations in the close environment. The route to the locations is displayed on the map, which supports a healthy lifestyle as extra steps can be accumulated.

### Qualitative assessment

One-on-one semi-structured interviews were conducted directly after using the KIJANI app. A previously generated interview guide including mostly open-ended questions was used (Table 1). The interview guide included “warm-up” questions to establish a good relationship with the participants and to inquire about their attitudes toward smartphone use, digital games, as well as PA. The main part of the interview related to the participants’ opinion on the KIJANI app. All interviews were conducted by the first author, who had already sufficient experience in research with children and adolescents (female, PhD in Health Science). The interviewer was not familiar with the participants prior to study initiation. Study participants did know that the researcher was doing these interviews in the TU Munich with the aim to further improve the KIJANI app. All interviews were conducted in person, were audio-recorded, and transcribed verbatim. Data collection proceeded until data saturation was achieved. Data saturation was reached after 22 interviews, when recurring themes were observed across various interviews, accompanied by a decreased emergence of new themes during analysis (inductive thematic saturation).

**Table 1.** Interview guide semi-structured interview.

	Topic	Main question	Detail question
Introduction	Attitude cell phone use	What do you normally use your cell phone for?	How much time do you spend on your cell phone?
	Attitude digital games	What do you think of mobile games?	Do you play any other games? Which ones? Have you played a similar game before?
	Attitude physical activity	What is your attitude towards sport and exercise?	Do you have any sports hobbies? Which ones? How do you feel when you play sports? Are you outside a lot? How do you get around/go to school/come here?
Main part	Evaluation KIJANI	How did you like the game KIJANI?	What would you change about the game? What did you (particularly) like about it? Are you satisfied with your building? Do you need anything else?
	Longevity of KIJANI	Can you imagine playing the game in the long term?	Would you like to continue playing the game with friends? When would you play the game?
	Activity promotion KIJANI	Do you think KIJANI has an impact on your activity behavior?	Does the game motivate you to walk to the game locations and take more steps in everyday life? Would you collect extra steps to earn coins for your buildings?
End	Possibility for own ideas	Is there anything else you would like to add?	

### Assessment of activity-related self-efficacy

Youth activity-related self-efficacy is defined “as a youth's belief in his/her capability to participate in PA and to choose PA despite existing barriers.”^21^ Activity-related self-efficacy was the only psychological factor consistently identified as a positive correlate and determinant of PA behavior in children and adolescents in several systematic reviews.^22–24^ Activity-related self-efficacy was assessed with the German version of the Physical Activity Self-efficacy Scale.^25^ The scale measures activity-related self-efficacy with six items and activity-related social support from family and friends via two items. Participants responded on a five-point Likert-type scale ranging from 1 (“Disagree a lot”) to 5 (“Agree a lot”), whereby higher values reflected higher activity-related self-efficacy. The questionnaire showed good internal consistency on the individual level and excellent on the class level. Reliability and validity of the German version were shown in a study with 454 German school children.^26^

### Enjoyment of physical activity

Enjoyment of PA was evaluated using the short version of the Physical Activity Enjoyment Scale (PACES-S). The PACES-S is a measurement tool that is frequently used in intervention research to measure the enjoyment of PA, as it is closely associated with adherence and compliance to PA.^27^ The PACES-S includes four items (i.e. “I enjoy it,” “I find it pleasurable,” “It is very pleasant,” “It feels good”). Items were answered on a five-point Likert scale ranging from (1) strongly disagree to (5) strongly agree, whereby higher values reflected greater levels of enjoyment. The German version of the PACES-S is reliable and valid for use with children and adolescents.^28^ The short version of the questionnaire showed comparable measurement properties to the long version of the PACES.^29^

### User experience

The User Experience Questionnaire (UEQ) was used to evaluate the subjective experience with the KIJANI app.^30^ User experience combines various aspects that are important for the subjective evaluation of a product including usability aspects, joy of use, as well as aesthetic design. The UEQ consists of 26 bipolar terms (pairs of opposites), which are evaluated over seven levels. The items consist of the following six pairs: attractiveness, perspicuity, efficiency, dependability, stimulation, and novelty. The questionnaire showed high reliability and construct validity.^31^

### Data analysis

Participants’ characteristics as well as values on activity-related self-efficacy, enjoyment of PA, and user experience are presented as mean ± standard deviation. Normal distribution was tested with the Shapiro–Wilk test. Depending on data distribution, differences between girls and boys were calculated with unpaired T-test or Mann–Whitney Test, as appropriate. Analysis was performed with RStudio version 4.1.2, with the level of significance set to two-sided p-values <0.05.

Data analysis of the semi-structured interviews was performed using the qualitative content analysis by Kuckarzt.^32,33^ After transcription of the interviews, categories were generated using an inductive-deductive approach, whereby category development was guided by a systematic reduction process during data analysis. Text sequences were placed in suitable categories, and thereby codes were generated. The interviews were evaluated by the respective interviewer and checked by a second person of the research team. Uncertainties concerning the coding scheme were discussed with the research team and homogeneously adjusted. The Software MAXQDA was used for data management.

## Results

Overall, 22 participants (12.6 ± 1.7 years, 6 girls) were included in the study. Detailed characteristics of the study population are displayed in Table 2.

**Table 2.** Study participants.

Characteristics	All (*n* = 22)	Boys (*n* = 16)	Girls (*n* = 6)	*P*-value*
Age (years)	12.6 ± 1.7	16.2 ± 1.7	12.3 ± 2.1	0.735
Activity-related self-efficacy	3.56 ± 0.95	3.51 ± 1.09	3.71 ± 0.46	0.671
Enjoyment of physical activity	3.81 ± 0.95	3.69 ± 1.05	4.13 ± 0.56	0.494^¥^
I enjoy it	3.86 ± 1.21	3.63 ± 1.26	4.50 ± 0.84	0.134^¥^
I find it pleasurable	3.77 ± 1.15	3.63 ± 1.15	4.17 ± 1.17	0.367^¥^
It is very pleasant	3.73 ± 1.01	3.63 ± 1.20	4.00 ± 0.63	0.641^¥^
It feels good	3.86 ± 1.04	3.88 ± 1.15	3.83 ± 0.75	0.747^¥^
User experience				
Attractiveness	1.78 ± 1.35	1.48 ± 1.47	2.58 ± 0.31	0.087
Perspicuity	2.15 ± 0.77	2.08 ± 0.85	2.33 ± 0.56	0.504
Efficiency	0.67 ± 1.12	0.44 ± 1.04	1.29 ± 1.17	0.113
Dependability	1.21 ± 0.97	1.14 ± 1.06	1.38 ± 0.70	0.624
Stimulation	1.24 ± 1.34	1.08 ± 1.45	1.67 ± 0.96	0.370
Novelty	1.27 ± 1.16	1.02 ± 1.20	1.96 ± 0.73	0.090

*Note*: Values displayed as mean ± standard deviation. *P-value calculated with unpaired *t*-test and Mann–Whitney-U (^¥^) as appropriate.

### Qualitative results

Interviews ranged between 3.12 and 8.11 min in length. Five main categories were established: overall impression, weaknesses, ideas for improvement, group play, as well as impact of KIJANI on PA.

The basic attitudes toward smartphone usage, playing smartphone games, as well as PA were inquired at the beginning of the interviews. Thirteen participants stated that they use mobile games. Two boys stated that they only play sometimes while 11 children (2 female (f), 9 male (m)) reported to play mobile games frequently with screen times ranging between two to eight hours per day. Two girls had mobile games on their smartphones but noted that they do not actively use them. Three participants (1f, 2m) had a time limit of one hour to use digital games on their smartphones and two boys had their phone completely blocked for games. One boy did not have a smartphone and one boy claimed that he does not play on his smartphone only on the computer.

Most of the study participants (*n* = 15, 10m, 5f) did enjoy being physically active and do sports. The remaining seven participants said that they are too lazy (*n* = 2, m), it's too exhausting (*n* = 1, m), they would not voluntarily do sports (*n* = 1, f), or it depends on weather and mood conditions (*n* = 3, m).

*Overall impression*. The overall game concept was liked by the huge majority of study participants. Things participants said about KIJANI were: “I thought it was really good” (m, years (y)); “Well, I thought it was very cool. [..] It encouraged you to walk more so that you could collect these points and that you could collect stones, for example, gold“ (f, 16 years); “I just think it's a good concept. Because there are a lot of people who don't exercise” (m, 14 years). Two participants mentioned that they especially like the graphic of the game (m). All but two participants wanted to download the KIJANI app once it was officially released, one participant was not yet sure without mentioning any particular reason (m) and one male participant was not at all interested in mobile games. Whilst some participants actively stated that they liked the fact that KIJANI can only be played outside and only at predefined play locations, some others wished “that you can play it at home as well” (m, 12 years). Several participants asked if play locations will be available around their hometowns, and thus showed interest in continuing to play KIJANI privately.

*Weaknesses*. Some participants identified weaknesses in the utilization of the KIJANI app. According to one participant (m, 12 years), “[…] it was a bit unrealistic. People could just walk through the buildings.” Another male participant (15 years) had concerns that “sometimes there are some bugs, where you simply can't place any blocks or can't remove them.” A 10-year-old boy mentioned, “I found it a bit confusing because the blocks appear to be on the ground. But when you crouch down, you see that there's still empty space below.” In addition, concerns regarding the GPS accuracy were mentioned, as the location of blocks varies a bit depending on the GPS (m). Hesitations were also expressed about playing KIJANI during winter when it is cold or wet outside (m).

Two boys did express doubts about the incentive of KIJANI: “If I reach the 10,000 steps now, then there is no goal anymore” (m, 15 years).

*Ideas for improvement*. A huge number of 14 participants had the idea to include more and a greater variety of blocks in KIJANI. Many different wishes concerning blocks were stated, ranging from blocks to build up proper houses including doors and windows, which can be opened and closed, as well as fences, and roof tiles. One boy (12 years) had the idea that “[…] it would be cool if you could also build stairs that you could then climb in the game.” In addition, house furnishing was stated several times including beds, chairs, and cupboards. Different textures were wished for like iron, marble, concrete, and different kinds of wood. Some participants also wanted to have blocks in different colors. Other participants had the idea to include plants or even animals. To make KIJANI more interesting over the long run, one girl (12 years) had the idea that “if you can't load them [updates] all immediately, that there are always updates where you can make two or three more blocks.” Also, several opportunities concerning the features of the blocks were mentioned. According to one male participant (14 years) KIJANI should “bring more physics into play.” He further explained that blocks should have some functions relatable to its real world function. For example, three boys (12 years, 12 years, 14 years) wanted, that “the water shouldn't be a block, it should flow.” Also, two boys (12 years, 14 years) wanted sand to be falling down when placed in the game.

Another idea concerning the improvement of KIJANI was the possibility to change the gaming environment to virtual worlds. “So maybe two game modes, one here in the real world. And then maybe a science fiction world” (m, 15 years). Another participant had the same idea with “the possibility to change maps between real world and fiction world” (m, 12 years).

One 15-year-old male participant suggested the incorporation of monsters or similar features into the game. These entities would pose a threat to the created buildings, fostering a greater incentive for users to remain active and engaged (m). Another boy (15 years) came up with a similar idea: “You could add fighting so that you can battle each other. With other groups or even animals” (m, 15 years).

One male participant (14 years) had the idea to “maybe change the goals a bit or perhaps add more features so that it becomes a bit more exciting.”

Group play. All participants mentioned that they liked the feature of KIJANI to play together as a group. One girl (16 years) said: “I think it’s cool that you can play in a group because then you might get even more excited. And if one person says yes, I’m going to play and everyone says ah yes, then we'll play, so we'll build a house together or something like that.” While the majority embraced the idea of collective gameplay, one 10-year-old girl expressed concerns about other players potentially destroying her buildings. In contrast, a 14-year-old boy found the prospect of such challenges to be an additional source of engagement.

One participant proposed the idea of extending the gameplay experience by allowing users to play together online with virtual friends (m).

*Impact of KIJANI on physical activity*. The great majority of participants think that KIJANI would increase their level of PA in the long-term (*n* = 16, 10m, 6f). A 14-year old boy said: “I do believe that this can motivate many people, including me.” Another girl (13 years) answered the question concerning the impact of KIJANI on PA “Clearly yes. And I don't think it would be just for me, but also for others.” Four boys did not think that an app like KIJANI has the potential to motivate them to be more physically active. “I don't think so because others want to play video games, and they don't feel like going outside” (m, 10 years). Two boys think it might have an impact on others, but not really on themselves (12 years, 15 years). However, they could not further specify why this is the case.

### Quantitative results

**Figure 2.**
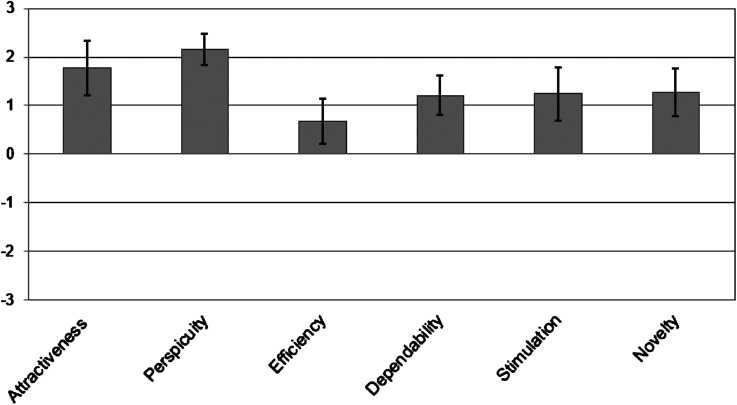
User experience questionnaire (means and standard deviation).

Activity-related self-efficacy scale (ranging from 0 to 5, higher values indicate higher activity-related self-efficacy) was 3.56 ± 0.95, whereby values did not differ between boys and girls (*P* = .671). The PACES-S (ranging from 0 to 5, higher values indicate higher PA enjoyment) was 3.81 ± 0.95, again with no difference between boys and girls (*P* = .494). The average UEQ (ranging from −3 to +3; whereby scores of 1–2 indicate excellent evaluation scale, scores above 0.8 indicate positive evaluation, scores between 0.8 and −0.8 neutral evaluation, scores <−0.8 report negative evaluation) was: attractiveness (1.78 ± 1.82), perspicuity (2.15 ± 0.68), efficiency (0.67 ± 1.25), dependability, (1.21 ± 0.93), stimulation (1.24 ± 1.78), and novelty (1.27 ± 1.34), see Figure 2. Values of the UEQ did not differ between genders.

## Discussion

KIJANI is a mobile game explicitly developed for activity promotion in children and adolescents with a special focus on PA in the outdoor setting as well as the creativity of users. This first multimethod evaluation highlights that overall, the game concept and realization were well received by the target group. Study participants did have various and creative ideas for further improvement of KIJANI, mostly focusing on the gaming experience including variety and function of blocks, as well as some utilization aspects. The big majority of study participants believed that KIJANI would help themselves and others to be more physically active in every-day life. However, so far, this can only be predicted on the basis of this evaluation. Results from this study like, for example, a greater variety of blocks will be integrated in the KIJANI app for further improvement. Afterwards, the next evaluation process will investigate the objective impact of KIJANI on PA behavior through a randomized controlled trial.

It has to be considered that the acceptance of health-oriented mobile apps like KIJANI depends on individual attitudes and behavior of the individual user. A previous study showed that acceptance was most often predicted by perceived usefulness, social influences, and attitude.^34^ This is especially true for existing attitudes toward digital games. A previous study examined why people play mobile location-based augmented reality games like Pokemon Go. They did report that previous experience and attitude toward these games have a huge influence on long-term usage.^35^ Our study cohort represents a variety of attitudes toward mobile gaming, PA, and sports, thereby reflecting the diversity in the population. Notably, participants who generally do not play smartphone games exhibited a lower level of acceptance toward KIJANI, indicating a clear association between pre-existing attitudes toward mobile gaming and the reception of health apps like KIJANI. This implies that digital interventions such as KIJANI appeal predominantly to a certain target group. Furthermore, our findings reveal a connection between favorable attitudes toward PA and sports and a more positive perception of KIJANI, particularly in relation to the fact that KIJANI can only be played outdoors in predefined locations. Understanding these relationships underscores the importance of individual attitudes and behaviors in the acceptance of health-oriented mobile applications like KIJANI.

The most frequent suggestions for improvement concerned more variety and possibilities in the game – including a variety of blocks, but also blocks that have a specific function or also game modifications. Especially participants, who frequently use other online games, had quite precise ideas, probably inspired by other online games.

A smartphone app called “MobileKids Monster Manor” (MKMM) employed a monster theme to boost PA in healthy adolescents. Similar to KIJANI, MKMM rewards real-world PA with gaming currency, in their case providing users with additional playtime. However, MKMM employs various incentives for long-term engagement, including positive peer pressure, rewards, and health competitions.^36^ In a school-based environment, this intervention increased PA.^37^ Enhancing KIJANI by incorporating additional incentives could be a valuable improvement, particularly considering feedback from our evaluation where some participants expressed a desire for incentives, especially in the long term.

“Zombies, Run!” is another smartphone app with the aim to increase cardiorespiratory fitness and PA levels in healthy young people through a gamification approach. The underlying concept is a bit different from KIJANI, as it consists of an 8-weeks training program. It is an immersive app where the training program is integrated into a storyline, prompting users to collect supplies and defend a town against zombies.^38^ Similar to MKMM, there is a constant requirement to stay active in the app over time, to prevent any negative consequences or destructions in the game. Creating a permanent, long-lasting incentive like for example animals or virtual creatures could also be a possibility to strengthen KIJANI, especially in the long term.

Mobile health apps aiming for long-term behavior change need users to stay active in the app over a prolongued time period. However, user retention with mobile apps is still a common issue in the field of mHealth. A previous study showed that sustained use of mHealth apps is closely linked to self-monitoring functions, which is also a feature included in the KIJANI app.^39^

However, overall it needs to be recognized, that long-term and sustainable behavior change toward a physically active lifestyle is highly complex and requires multifactorial approaches.^40^ Behavioral interventions promoting higher PA are often unsuccessful, especially having problems in maintaining the adherence of the target group in the long term.^41^ The opportunity to make prevention and health promotion attractive and age-appropriate through the use of digital media should be utilized. We have therefore taken up the idea of developing a digital activity promotion approach with a participatory evaluation process and hope that KIJANI could have an impact on the long-term activity behavior of at least part of the target group.

### Limitations

Several limitations should be considered when discussing the results of this study. Firstly, the gender distribution in our cohort is unequal, with very few girls included. Additionally, the time allocated for using KIJANI was limited to only 25 min in our study, meaning our findings primarily reflect participants’ first impressions, and extrapolation of these results to long-term use should therefore be treated with caution.

One-on-one interviews carry the risk of social desirability bias, where participants may provide responses perceived as socially acceptable rather than expressing their true opinions or behaviors. This could impact the reliability of our data and should be considered when interpreting the study's results. Furthermore, activity-related self-efficacy and physical activity enjoyment were only measured once, post-intervention, and thus cannot provide information on the impact of the KIJANI intervention.

## Conclusion

In conclusion, our study indicates that KIJANI, a mobile game focusing on PA promotion in children and adolescents, has been well-received by the target audience. With the insights gained in this study, a further step has been taken in the participatory development of KIJANI and will guide further improvements to the app. Findings of this study will be incorporated to enhance KIJANI and therefore physical activity levels of children and adolescents. In conclusion, the insights derived from this research are transferable, offering valuable guidance for the development of future games focused on promoting PA.
